# Non contiguous-finished genome sequence and description of *Bacillus massiliosenegalensis* sp. nov.

**DOI:** 10.4056/sigs.3496989

**Published:** 2013-06-05

**Authors:** Dhamodharan Ramasamy, Jean-Christophe Lagier, Aurore Gorlas, Didier Raoult, Pierre-Edouard Fournier

**Affiliations:** 1 Aix-Marseille Université, URMITE, Faculté de médecine, Marseille, France

**Keywords:** *Bacillus massiliosenegalensis*, genome, culturomics, taxono-genomics

## Abstract

*Bacillus massiliosenegalensis* strain JC6^T^ sp. nov. is the type strain of *Bacillus massiliosenegalensis* sp. nov., a new species within the genus *Bacillus*. This strain was isolated from the fecal flora of a healthy Senegalese patient. *B. massiliosenegalensis* is an aerobic Gram-positive rod-shaped bacterium. Here we describe the features of this organism, together with the complete genome sequence and annotation. The 4,981,278-bp long genome comprises a 4,957,301-bp chromosome and a 23,977-bp plasmid. The chromosome contains 4,925 protein-coding and 72 RNA genes, including 4 rRNA genes. The plasmid contains 29 protein-coding genes.

## Introduction

*Bacillus massiliosenegalensis* strain JC6^T^ (= CSUR P151 = DSM 25957) is the type strain of *B. massiliosenegalensis* sp. nov., a new species within the genus *Bacillus*. This bacterium is a Gram-positive, aerobic, catalase-positive and indole-negative bacillus that was isolated from the stool of a healthy Senegalese patient as part of a study aimed at individually cultivating all human enteric bacterial species [1,2].

Currently, bacterial taxonomy relies on a combination of various genetic and phenotypic criteria. However, the three main genetic criteria that are used, including 16S rRNA gene-based phylogeny and nucleotide similarity [3,4], DNA-DNA hybridization [5] and G+C content suffer significant drawbacks and their cutoffs are not applicable to all genera and species. Over recent years, the introduction of high-throughput genome sequencing and proteomic analyses [6] provided a source of exhaustive information about characterized bacterial isolates. Such data may now be included among the criteria used for taxonomic identification. We recently proposed to use a polyphasic approach to describe new bacterial taxa that is based on their genome sequence, MALDI-TOF spectrum and main phenotypic characteristics [7-25].

Here we present a summary classification and a set of features for *B. massiliosenegalensis* sp. nov. strain JC6^T^ together with the description of the complete genomic sequencing and annotation. These characteristics support the creation of the species *B. massiliosenegalensis*.

The genus *Bacillus* (Cohn 1872) was created in 1872 [26] and currently consists of mainly Gram-positive, motile, and spore-forming bacilli. Currently, 173 *Bacillus* species and 4 subspecies are validly published [27]. Members of the genus *Bacillus* are ubiquitous bacteria, mostly isolated from environmental sources. However, several species are associated with humans, either as pathogens or commensals [28].

## Classification and features

A stool sample was collected from a healthy 16-year-old male Senegalese volunteer patient living in Dielmo (rural village in the Guinean-Sudanian zone in Senegal), who was included in a research protocol. Written assent was obtained from this individual. No written consent was needed from his guardians for this study because he was older than 15 years old (in accordance with the previous project approved by the Ministry of Health of Senegal and the assembled village population and as published elsewhere [29].)

Both this study and the assent procedure were approved by the National Ethics Committee of Senegal (CNERS) and the Ethics Committee of the Institut Fédératif de Recherche IFR48, Faculty of Medicine, Marseille, France (agreement numbers 09-022 and 11-017). Several other new bacterial species were isolated from this specimen using various culture conditions, including the recently described *Alistipes senegalensis*, *Alistipes timonensis*, *Anaerococcus senegalensis*, *Bacillus timonensis*, *Clostridium senegalense*, *Peptoniphilus timonensis* and *Paenibacillus senegalensis*, *Herbaspirillum massiliense, Kurthia massiliensis, Brevibacterium senegalense, Aeromicrobium massilense, Cellulomonas massiliensis, Senegalemassilia anaerobia, Peptoniphilus obesi, Peptoniphilus senegalensis, Enterobacter massiliensis, Alistipes obesi, Peptoniphilus grossensis, Brevibacillus massiliensis* [7-25].

**Figure 1:**
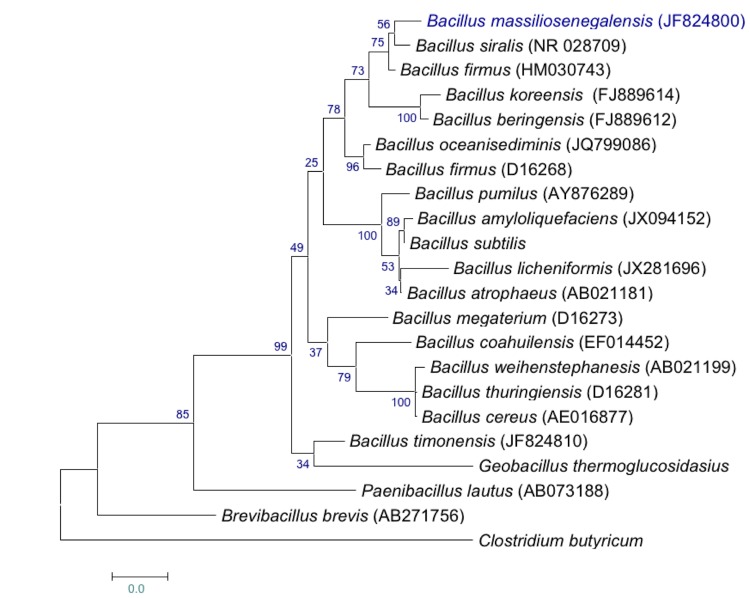
Phylogenetic tree showing the position of *Bacillus massiliosenegalensis* strain JC6^T^ relative to other type strains within the *Bacillus* genus. GenBank accession numbers are indicated in parentheses. Sequences were aligned using CLUSTALW, and phylogenetic inferences obtained using the maximum-likelihood method within the MEGA software. Numbers at the nodes are percentages of bootstrap values obtained by repeating the analysis 500 times to generate a majority consensus tree. *Clostridium butyricum* was used as outgroup. The scale bar represents a 2% nucleotide sequence divergence.

The fecal specimen was conserved at -80°C after collection. Strain JC6^T^ was (Table 1) was isolated in January 2011 by cultivation on 5% sheep blood-enriched Brain Heart infusion (BHI) agar (Becton Dickinson, Heidelberg, Germany). The strain exhibited a 97.3% nucleotide sequence similarity with *B. siralis* (Pettersson *et al.* 2000), the phylogenetically closest *Bacillus* species (Figure 1).

**Table 1:** Classification and general features of *Bacillus massiliosenegalensis* strain JC6^T^ according to the MIGS recommendations [30]

**MIGS ID**	**Property**	**Term**	**Evidence code^a^**
	Current classification	Domain *Bacteria* Phylum *Firmicutes* Class *Bacilli* Order *Bacillales* Family *Bacillaceae* Genus *Bacillus* Species *Bacillus massiliosenegalensis* Type strain JC6^T^	TAS [31] TAS [32-35] TAS [36,37] TAS [32,38] TAS [32,39] TAS [32,40,41] IDA IDA
	Gram stain	Positive	IDA
	Cell shape	Rod	IDA
	Motility	Motile	IDA
	Sporulation	Sporulating	IDA
	Temperature range	Mesophile	IDA
	Optimum temperature	30°C	IDA
MIGS-6.3	Salinity	Growth in BHI medium + 5% NaCl	IDA
MIGS-22	Oxygen requirement	Aerobic	IDA
	Carbon source	Unknown	NAS
	Energy source	Unknown	NAS
MIGS-6	Habitat	Human gut	IDA
MIGS-15	Biotic relationship	Free living	IDA
MIGS-14	Pathogenicity Biosafety level Isolation	Unknown 2 Human feces	NAS
MIGS-4	Geographic location	Senegal	IDA
MIGS-5	Sample collection time	September 2010	IDA
MIGS-4.1	Latitude	13.7167	IDA
MIGS-4.1	Longitude	-16.4167	IDA
MIGS-4.3	Depth	Surface	IDA
MIGS-4.4	Altitude	51 m above sea level	IDA

Evidence codes - IDA: Inferred from Direct Assay; TAS: Traceable Author Statement (i.e., a direct report exists in the literature); NAS: Non-traceable Author Statement (i.e., not directly observed for the living, isolated sample, but based on a generally accepted property for the species, or anecdotal evidence). These evidence codes are from the Gene Ontology project [42]. If the evidence is IDA, then the property was directly observed for a live isolate by one of the authors or an expert mentioned in the acknowledgements.

**Figure 2:**
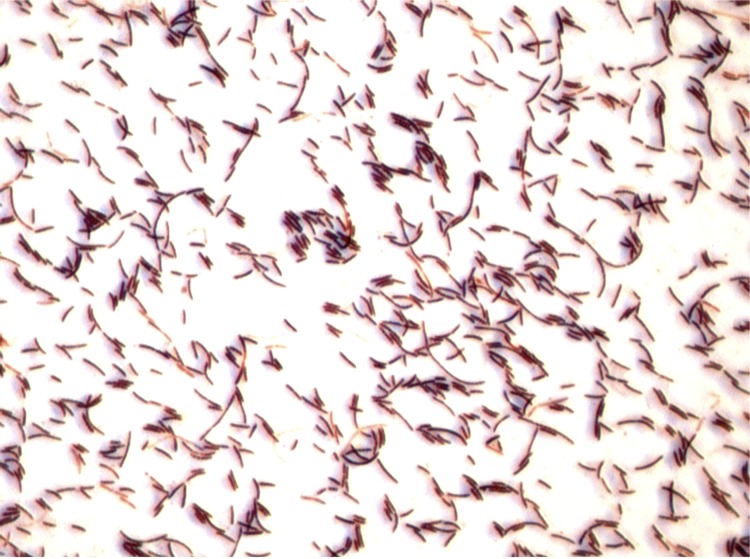
Gram staining of *B. massiliosenegalensis* strain JC6^T^

**Figure 3:**
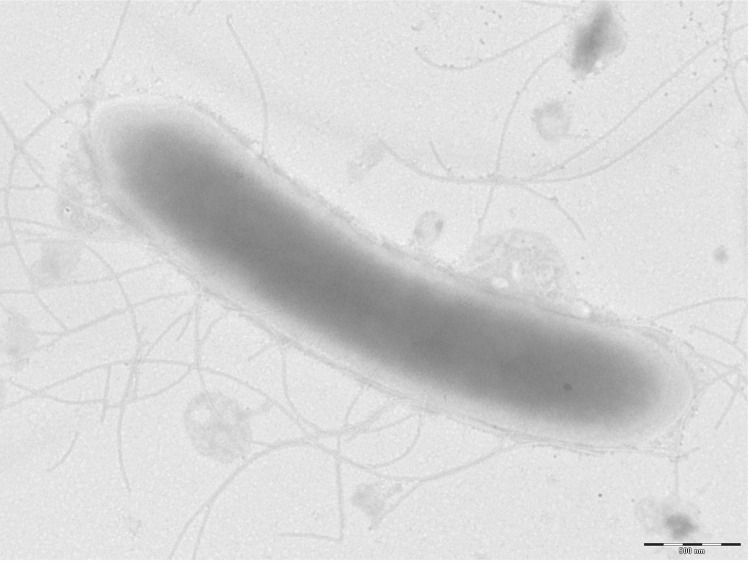
Transmission electron microscopy of *B. massiliosenegalensis* strain JC6^T^, using a Morgani 268D (Philips) at an operating voltage of 60kV. The scale bar represents 500 nm.

Different growth temperatures (25, 30, 37, 45°C) were tested. Growth occurred between 25°C and 45°C, and the optimal growth was observed at 30°C. Colonies were translucent and 2 mm in diameter on blood-enriched Columbia agar. Growth of the strain was tested under anaerobic and microaerophilic conditions using GENbag anaer and GENbag microaer systems respectively (BioMérieux) in the presence of air with or without 5% CO_2_. Growth was achieved in aerobic condition (with or without CO_2_), and weak growth was observed in microaerophilic and anaerobic conditions. Gram staining showed a rod-shaped Gram-positive bacterium (Figure 2). The motility test was positive by means of peritrichous flagella. Cells have a mean diameter of 0.65 µm and a mean length of 3.076 µm in electron microscopy (Figure 3).

Strain JC6^T^ exhibited catalase activity but not oxidase activity. Using the API 50CH system, we observed positive reactions for aesculin, D-cellobiose, D-glucose, D-maltose, N-acetyl-glucosamine and D-trehalose. Using the API ZYM system, a positive reaction was observed for α-glucosidase and weak reactions were observed for alkaline phosphatase, esterase lipase, valine arylamidase and trypsin. Using the API 20E system, a positive reaction was observed for nitrate reduction and negative reactions were observed for indole production and urease. *B. massiliosenegalensis* is susceptible to amoxicillin, ceftriaxone, imipenem, trimethoprim/sulfamethoxazole, gentamicin, ciprofloxacin, rifampicin and vancomycin, but resistant to metronidazole and erythromycin. The differential phenotypic characteristics with other *Bacillus* species are summarized in Table 2.

**Table 2:** Differential characteristics of *B. massiliosenegalensis* strain JC6^T^, *B. timonensis* strain MM10403188^T^, *B. amyloliquefaciens* strain FZB42, *B. siralis* strain 171544 ^T^ and *B. thuringiensis* strain BMB171

**Properties**	*B. massiliosenegalensis*	*B. timonensis*	*B. amyloliquefaciens*	*B. siralis*	*B. thuringiensis*
Cell diameter (µm)	0.65	0.66	0.8	0.5 to 0.8	1.0
Oxygen requirement	aerobic	aerobic	aerobic	aerobic	facultative anaerobic
Pigment production	–	–	–	+	–
Gram stain	+	–	+	+	+
Salt requirement	+	+	+	+	
Motility	+	+	+		–
Endospore formation	+	+	+	+	+
**Production of**					
Acid phosphatase	w	na	+	na	+
Catalase	+	–	+	–	+
Oxidase	–	+	+	+	+
Nitrate reductase	+	na	+	+	+
Urease	-	na	–	na	+
β-galactosidase	na	+	v	na	–
N-acetyl-glucosamine	+	+	+	na	+
**Acid from**					
L-Arabinose	–	+	+	–	na
Ribose	–	–	+	–	+
Mannose	–	–	+	–	+
Mannitol	–	–	+	–	+
Sucrose	–	–	+	–	v
D-glucose	+	–	+	–	+
D-fructose	–	–	+	–	+
D-maltose	+	–	+	–	+
D-lactose	–	+	+	–	+
**Hydrolysis of**					
Gelatin	–	–	+	+	+
**G+C content (mol%)**	37.6	37.3	46.48	na	35.18
**Habitat**	human gut	human gut	Soil	silage	soil

na = data not available; w = weak, v = variable reaction

**Figure 4:**
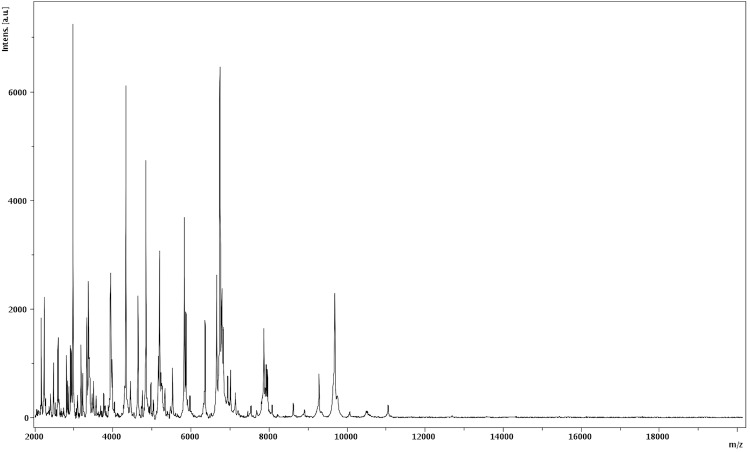
Reference mass spectrum from *B. massiliosenegalensis* strain JC6^T^. Spectra from 12 individual colonies were compared and a reference spectrum was generated.

**Figure 5:**
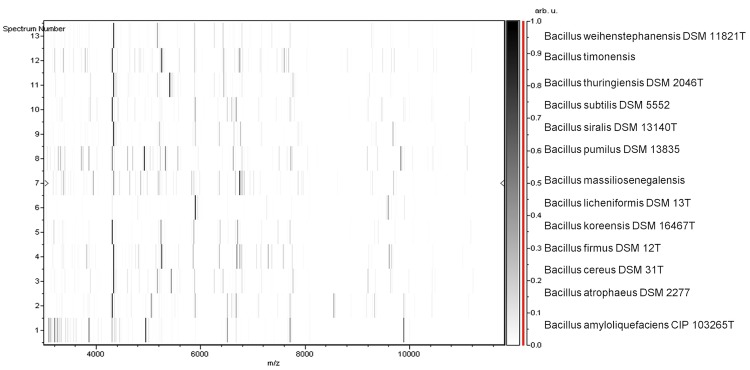
Gel view comparing *B. massiliosenegalensis* sp. nov strain JC6^T^ and other *Bacillus* species. The gel view displays the raw spectra of loaded spectrum files arranged in a pseudo-gel like look. The x-axis records the m/z value. The left y-axis displays the running spectrum number originating from subsequent spectra loading. The peak intensity is expressed by a Gray scale scheme code. The color bar and the right y-axis indicate the relation between the color withwhich a peak is displayed and the peak intensity in arbitrary units. Displayed species are indicated on the left.

Matrix-assisted laser-desorption/ionization time-of-flight (MALDI-TOF) MS protein analysis was carried out as previously described [8,43] using a Microflex spectrometer (Bruker Daltonics, Germany). Spectra were compared with the Bruker database that contained the main spectra from 3,769 bacteria including 129 spectra from 98 validly named *Bacillus* species. No significant score was obtained, thus suggesting that our isolate was not a member of a known species. We incremented our database with spectrum from strain JC6^T^ (Figure 4). Finally, the gel view allows us to highlight the spectra differences with other *Bacillus* genera members Figure 5.

## Genome sequencing information

### Genome project history

The organism was selected for sequencing on the basis of its phylogenetic position and 16S rRNA similarity to other members of the genus *Bacillus*, and is part of a study of the human digestive flora aiming at isolating all bacterial species within human feces [1,2]. It was the 268^th^ genome of a *Bacillus* species, and the first genome of *B. massiliosenegalensis* sp. nov. The Genbank accession number is CAHJ00000000 and consists of 102 contigs. Table 3 shows the project information and its association with MIGS version 2.0 compliance [44].

**Table 3:** Project information

**MIGS ID**	**Property**	**Term**
MIGS-31	Finishing quality	High-quality draft
MIGS-28	Libraries used	454 GS shotgun and paired-end 3- kb libraries
MIGS-29	Sequencing platform	454 GS FLX Titanium
MIGS-31.2	Sequencing coverage	54.25×
MIGS-30	Assemblers	Newbler version 2.5.3
MIGS-32	Gene calling method	PRODIGAL
	Genbank Date of Release Gold ID	June 1, 2012 Gi18838
	NCBI project ID	CAHJ00000000
MIGS-13	Project relevance	Study of the human gut microbiome

### Growth conditions and DNA isolation

*B. massiliosenegalensis* sp. nov. strain JC6^T^, CSUR P151, DSM 25957, was grown aerobically on 5% sheep blood-enriched BHI agar at 37°C. Five petri dishes were spread and cultivated bacteria were resuspended in 3×100µl of G2 buffer (EZ1 DNA Tissue kit, Qiagen). A first mechanical lysis was performed by glass powder on the Fastprep-24 device (MP Biomedicals, USA) using 2×20 seconds cycles. DNA was then treated with 2.5 µg/µL lysozyme for 30 minutes at 37°C, and extracted using a BioRobot EZ 1 Advanced XL (Qiagen). The DNA was then concentrated and purified using a Qiamp kit (Qiagen). The yield and the concentration was measured by the Quant-it Picogreen kit (Invitrogen) on the Genios_Tecan fluorometer at 36.8 ng/µl.

### Genome sequencing and assembly

Both a shotgun and a 3kb paired-end libraries were constructed. The shotgun library was constructed with 500 ng of DNA as described by the manufacturer with the GS Rapid library Prep kit (Roche). For the paired-end library, 5µg of DNA was mechanically fragmented on the Hydroshear device (Digilab, Holliston, MA,USA) with an enrichment size at 3-4kb. The DNA fragmentation was visualized using an Agilent 2100 BioAnalyzer on a DNA labchip 7500 with an optimal size of 3.2kb. The library was constructed according to the 454 GS FLX Titanium paired-end protocol (Roche). Circularization and nebulization were performed and generated a pattern with an optimal at 555 bp. After PCR amplification through 17 cycles followed by double size selection, the single stranded paired-end library was then quantified on the Quant-it Ribogreen kit (Invitrogen) on the Genios Tecan fluorometer at 21pg/µL. The library concentration equivalence was calculated at 6.94E+07 molecules/µL. Libraries were stored at -20°C until further use.

The shotgun library was clonally amplified with 2 cpb in 4 emPCR reactions, whereas the 3kb paired-end library was amplified with 1cpb in 9 emPCR reactions and 0.5 cpb in 2 emPCR with the GS Titanium SV emPCR Kit (Lib-L) v2 (Roche). The yield of the shotgun emPCR reaction was 8.12%, and those of the 2 kinds of paired-end emPCR reactions were 7.8% and 11.2%, respectively. Such results were in the 5-20% quality range expected from the Roche procedure. For sequencing, the shotgun and paired-end libraries were loaded onto the 1/2 region and 4 1/4 regions of a PTP Picotiterplate 70×75 (Roche), respectively. The sequencing reactions were performed using a GS FLX Titanium sequencing kit XLR70 (Roche). The run was performed overnight and then analyzed on the cluster through the gsRunBrowser and Newbler assembler (Roche).

A total of 969,014 passed filter wells were obtained and generated 274 Mb of sequences with a length average of 286 bp. These sequences were assembled using Newbler with 90% identity and 40 bp as overlap. The final assembly identified 31 scaffolds and 129 large contigs (>1,500 bp), generating a genome size of 5.05 Mb, which corresponds to a 54.25-fold coverage.

### Genome annotation

Open Reading Frames (ORFs) were predicted using Prodigal [45] with default parameters. However, the predicted ORFs were excluded if they spanned a sequencing gap region. The predicted bacterial protein sequences were searched against the GenBank [46] and Clusters of Orthologous Groups (COG) databases using BLASTP. tRNAs and ribosomal RNAs were predicted using the tRNAScanSE [47] and RNAmmer [48] tools, respectively. Lipoprotein signal peptides and numbers of transmembrane helices were predicted using SignalP [49] and TMHMM [50], respectively. ORFans were identified if their BLASTP *E*-value was lower than 1e-03 for alignment length greater than 80 amino acids. If alignment lengths were smaller than 80 amino acids, we used an *E*-value of 1e-05. Such parameter thresholds have already been used in previous works to define ORFans. Artemis [51] was used for data management and DNA Plotter [52] was used for visualization of genomic features. The alignment tool, Mauve, was used for multiple genomic sequence alignment [53]. To estimate the mean level of nucleotide sequence similarity at the genome level between *B. massiliosenegalensis* and 5 other Bacillus genomes (Table 4), orthologous proteins were detected using the Proteinortho [54] and we compared genomes two by two and determined the mean percentage of nucleotide sequence identity among orthologous ORFs using BLASTn.

**Table 4:** List of *Bacillus* species genomes used for genomic comparison

Species	Strain	Genome accession number	Genome Size (Mb)	G+C content %
*B. massiliosenegalensis*	JC6^T^	CAHJ00000000	4,981,278	37.60
*B. timonensis*	MM10403188^T^	CAET00000000	4,632,049	37.30
*B. cereus*	03BB102	NC_012472	5,269,628	35.40
*B. licheniformis*	ATCC 14580	NC_006270	4,222,597	46.20
*B. subtilis*	168	NC_000964	4,215,606	43.50
*B. thuringiensis*	BMB171	NC_014171	5,330,088	35.30

## Genome properties

**Figure 6:**
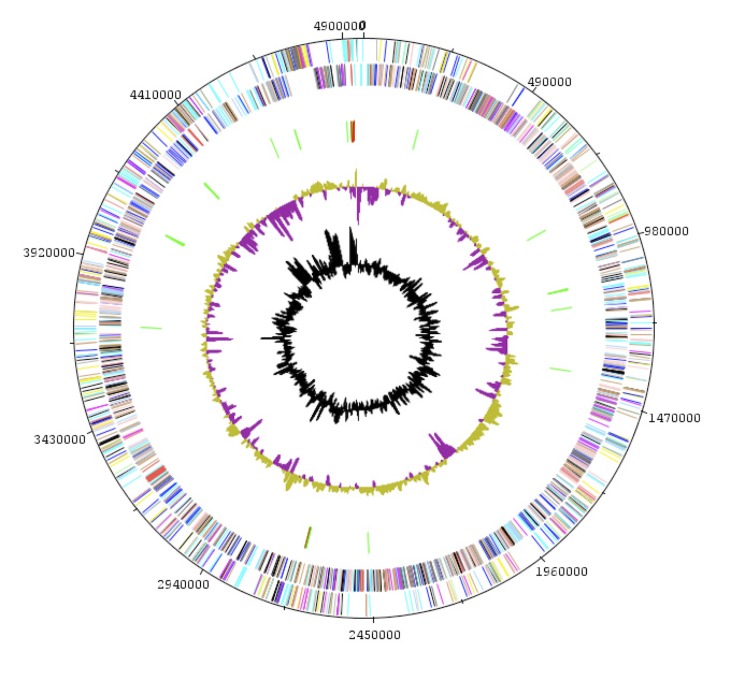
Graphical circular map of the chromosome. From outside to the center: Genes on the forward strand (colored by COG categories), genes on the reverse strand (colored by COG categories), RNA genes (tRNAs green, rRNAs red), GC content, and GC skew.

The genome is 4,981,278 bp long (chromosome: 4,957,301 bp, plasmid: 23,977 bp) with a GC content of 37.60% (Figure 6 and Table 5). Of the 4,997 predicted chromosomal genes, 4,925 were protein-coding genes and 72 were RNAs. A total of 3,554 genes (72.16%) were assigned a putative function. ORFans accounted for 338 (6.86%) of the genes identified. The remaining genes were annotated as hypothetical proteins. The properties and statistics of the genome are summarized in Tables 5 and 6. The distribution of genes into COGs functional categories is presented in Table 6. The 23,977 bp-long plasmid contains 29 protein-coding genes.

**Table 5:** Nucleotide content and gene count levels of the chromosome

**Attribute**	**Value**	**% of total^a^**
Genome size (bp)	4,957,301	
DNA coding region (bp)	4,222,509	85.17
DNA G+C content (bp)	1,863,945	37.6
Total genes	4,997	100
RNA genes	72	1.44
Protein-coding genes	4,925	98.55
Genes with function prediction	3,554	72.16
Genes assigned to COGs	3,142	63.79
Genes with peptide signals	223	4.52
Genes with transmembrane helices	1,329	26.59

^a^ The total is based on either the size of the genome in base pairs or the total number of protein coding genes in the annotated genome

**Table 6:** Number of genes associated with the 25 general COG functional categories

**Code**	**Value**	**% of total**^a^	**Description**
J	167	3.39	Translation
A	0	0	RNA processing and modification
K	252	5.11	Transcription
L	171	3.47	Replication, recombination and repair
B	0	0	Chromatin structure and dynamics
D	37	0.75	Cell cycle control, mitosis and meiosis
Y	0	0	Nuclear structure
V	82	1.66	Defense mechanisms
T	146	2.96	Signal transduction mechanisms
M	162	3.28	Cell wall/membrane biogenesis
N	23	0.46	Cell motility
Z	0	0	Cytoskeleton
W	0	0	Extracellular structures
U	32	0.64	Intracellular trafficking and secretion
O	105	2.13	Posttranslational modification, protein turnover, chaperones
C	238	4.83	Energy production and conversion
G	186	3.77	Carbohydrate transport and metabolism
E	262	5.31	Amino acid transport and metabolism
F	72	1.46	Nucleotide transport and metabolism
H	112	2.27	Coenzyme transport and metabolism
I	85	1.72	Lipid transport and metabolism
P	227	4.61	Inorganic ion transport and metabolism
Q	29	0.58	Secondary metabolites biosynthesis, transport and catabolism
R	407	8.26	General function prediction only
S	347	7.04	Function unknown
-	412	8.36	Not in COGs

^a^ The total is based on the total number of protein coding genes in the annotated genome

## Comparison with the genomes from other *Bacillus* species

**Figure 7:**
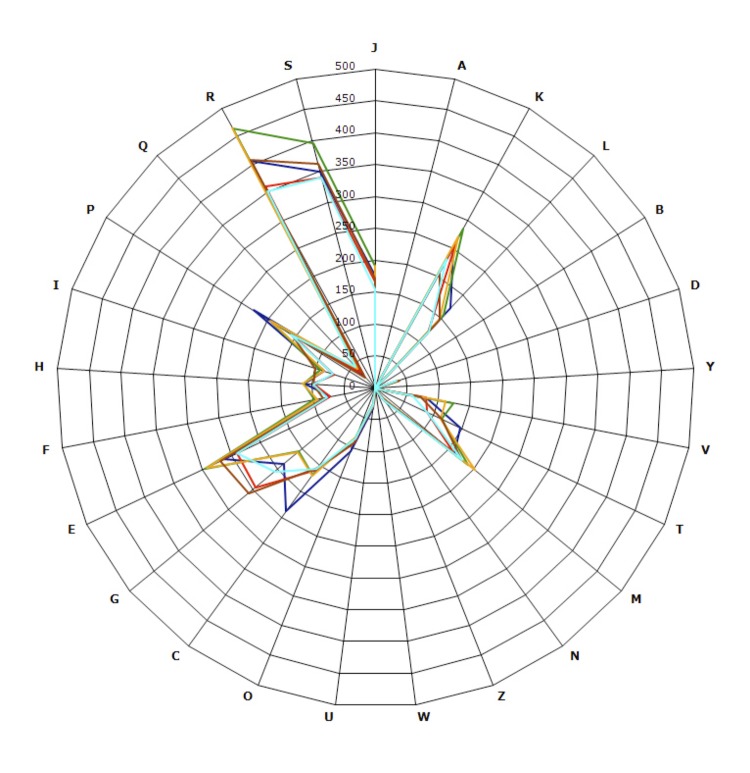
Distribution of functional classes of predicted genes on *B. massiliosenegalensis* (dark blue), *B. cereus* (green), *B. licheniformis* (red), *B. thuringiensis* (yellow) *B. timonensis* (brown) and *B. subtilis* (light blue) chromosomes according to the clusters of orthologous groups of proteins.

Currently, more than 50 complete genome sequences are available for *Bacillus* species. Here, we compared the genome sequence of *B. massiliosenegalensis* strain JC6^T^ with those of another 5 *Bacillus* species (Table 3). The genome sequence of *B. massiliosenegalensis* (4.98 Mb) is larger than those from *B. cereus*, *B. licheniformis*, *B. subtilis*, and *B. timonensis* ( 4.89, 4.22, 4.21, and 4.63 Mb, respectively) but smaller than *B. thuringiensis* (5.33 Mb). The G+C content of *B. massiliosenegalensis* is higher than *B. cereus, B. thuringiensis* and *B. timonensis* (37.6, 35.4, 35.3 and 37.3%, respectively) but lower than *B. licheniformis* and *B. subtilis* (46.2 and 43.5%, respectively). *B. massiliosenegalensis* has more predicted genes than *B. licheniformis*, *B. subtilis*, and *B. timonensis* (4,895, 4,295, 4,226, 4,610, respectively) but fewer genes than *B. thuringiensis* and *B. cereus* (5,341 and 5,331, respectively). However, the distribution of genes into COG categories was similar in all 6 compared genomes (Figure 7). In addition, B. *massiliosenegalensis* shared 2,022, 2,052, 2,009, 1,968 and 2,036 orthologous genes with *B.timonensis, B. cereus, B. licheniformis, B. subtilis and B. thuringiensis* respectively. The average nucleotide sequence identity ranged from 66.27 to 92.71% among the 6 *Bacillus* species, and from 67.05 to 70.42% between B. *massiliosenegalensis* and other *Bacillus* compared, thus confirming its new species status (Table 7). Finally, the plasmid genome of *B. massiliosenegalensis* is closely matching the *Bacillus cereus* Q1 plasmid (GenBank accession number CP000228).

**Table 7:** Genomic comparison of *B. massiliosenegalensis* with five other *Bacillus* species^†^.

Species	*B. massiliosenegalensis*	*B. timonensis*	*B. cereus*	*B. licheniformis*	*B. subtilis*	*B. thuringiensis*
*B. massiliosenegalensis*	**4,895**	2,022	2,052	2,009	1,968	2,036
*B. timonensis*	70.42	**4,610**	2,038	1,976	1,962	2,028
*B. cereus*	68.94	69.35	**5,331**	2,116	2,111	2,779
*B. licheniformis*	67.05	67.19	66.29	**4,295**	2,350	2,095
*B. subtilis*	67.70	67.93	67.28	74.01	**4,226**	2,087
*B. thuringiensis*	68.91	69.32	92.71	66.27	67.30	**5,341**

^†^The numbers of orthologous protein shared between genomes (above diagonal), average percentage similarity of nucleotides corresponding to orthologous protein shared between genomes (below diagonal) and the numbers of proteins per genome (bold).

## Conclusion

On the basis of phenotypic, phylogenetic and genome analysis, we formally propose the creation of *Bacillus massiliosenegalensis* sp. nov. which contains the strain JC6^T^. This strain has been found in Senegal.

### Description of *Bacillus massiliosenegalensis* sp. nov.

*Bacillus massiliosenegalensis* (mas.si.li.o.se.ne.gal.en′sis. L. gen. masc. n. *massiliosenegalensis*, contraction of the Latin names of Marseille and Senegal, where strain JC6^T^ was cultivated and collected, respectively.) *B. massiliosenegalensis* is an aerobic Gram-positive bacterium. Optimal growth is achieved aerobically and weak growth is observed under microaerophilic or anaerobic conditions. Growth occurs on axenic media between 25 and 45°C, with optimal growth observed at 37°C. Cells stain Gram-positive, are rod-shaped, endospore-forming, motile and have a mean diameter of 0.65 µm and a mean length of 3.076 µm. Peritrichous flagellae were observed. Colonies are translucent and 2 mm in diameter on blood-enriched BHI agar.

No oxidase activity detected. Negative for indole. Presence of catalase activity. Using the API 50CH system, positive reactions are observed for aesculin, D-cellobiose, D-glucose, D-maltose, N-acetyl-glucosamine and D-trehalose. Using the API ZYM system, a positive reaction is observed for α-glucosidase and weak reactions are observed for alkaline phosphatase, esterase lipase, valine arylamidase and trypsin. Using the API 20E system, a positive reaction is observed for nitrate reduction and a negative reaction is observed for urease. *B. massiliosenegalensis* is susceptible to amoxicillin, ceftriaxone, imipenem, trimethoprim/sulfamethoxazole, gentamicin, ciprofloxacin, rifampicin and vancomycin, but resistant to metronidazole and erythromycin.

The G+C content of the genome is 37.60%. The 16S rRNA and genome sequences are deposited in Genbank and EMBL under accession numbers JF824800 and CAHJ00000000, respectively. The type strain is JC6^T^ (= CSUR P151 = DSM 25957) was isolated from the fecal flora of a healthy Senegalese patient.
